# Dual diagnosis of TBI and SCI: an epidemiological study in the pediatric population

**DOI:** 10.3389/fneur.2023.1241550

**Published:** 2023-09-27

**Authors:** Joslyn Gober, Lauren T. Shapiro, Eduard Tiozzo, Nanichi A. Ramos Roldán, Cristina M. Brea, Katherine Lin, Adriana Valbuena

**Affiliations:** ^1^Department of Physical Medicine and Rehabilitation, University of Miami Miller School of Medicine, Miami, FL, United States; ^2^South Texas Veterans Health Care System and Department of Rehabilitation Medicine, University of Texas Health San Antonio, San Antonio, TX, United States

**Keywords:** dual diagnosis, Pediatrics, traumatic brain injury, spinal cord injury, epidemiology

## Abstract

**Introduction:**

Dual diagnosis (DD) with traumatic brain injury (TBI) and spinal cord injury (SCI) poses clinical and rehabilitation challenges. While comorbid TBI is common among adults with SCI, little is known about the epidemiology in the pediatric population. The primary objective of this study was to evaluate the prevalence of TBI among children in the United States hospitalized with SCI. Secondary objectives were to compare children hospitalized with DD with those with isolated SCI with regards to age, gender, race, hospital length of stay, and hospital charges.

**Methods:**

A retrospective analysis of hospital discharges among children aged 0–18 years occurring between 2016–2018 from U.S. hospitals participating in the Kids’ Inpatient Database. ICD-10 codes were used to identify cases of SCI, which were then categorized by the presence or absence of comorbid TBI.

**Results:**

38.8% of children hospitalized with SCI had a co-occurring TBI. While DD disproportionately occurred among male children (67% of cases), when compared with children with isolated SCI, those with DD were not significantly more likely to be male. They were more likely to be Caucasian. The mean age of children with DD (13.2 ± 5.6 years) was significantly less than that of children with isolated SCI (14.4 ± 4.3 years). DD was associated with longer average lengths of stay (6 versus 4 days) and increased mean total hospital charges ($124,198 versus $98,089) when compared to isolated SCI.

**Conclusion:**

Comorbid TBI is prevalent among U.S. children hospitalized with SCI. Future research is needed to better delineate the impact of DD on mortality, quality of life, and functional outcomes.

## Introduction

Neurological insults are a significant pediatric health issue, both nationally and internationally (1). While traumatic brain injuries (TBIs) are relatively common, traumatic spinal cord injuries (SCIs) are an uncommon cause of morbidity and mortality in children. However, when they occur, they represent a different challenge than SCI in adults (2, 3). Co-morbid traumatic brain injury (TBI) with spinal cord injury (SCI) may greatly impact patients’ rehabilitation courses and functional outcomes (4). Unfortunately, there remains a dearth of literature addressing the implications of these dual diagnoses (DD) in pediatric patients.

Prior retrospective reviews using large datasets, including the Kids’ Inpatient Database (KID), have elucidated demographic information regarding children who have sustained (isolated) TBIs and spinal injuries and have provided information regarding in-hospital mortality rates. Using the KID, Lu et.al identified 220,771 pediatric cases of TBI between 2006 and 2012, 66% of which occurred among boys. They reported a mean hospital length of stay of 5 days and an in-hospital mortality rate of 4% (5). Piatt used the KID to determine that the incidence of hospital admission for SCI among individuals aged 21 or younger was 24 per 1 million in 2009. That year, there were 2,139 cases of SCI identified in the dataset. 2.8% of those cases reportedly resulted in death during the hospitalization (6).

Prior research has also yielded information about the epidemiology of DD, though this has been better studied in adult populations. It is well-recognized that adults with SCI commonly have comorbid TBIs. The incidence of SCI in comatose patients is higher than the general trauma population (7). While there has been considerable variability in the estimated prevalence of DD, studies suggest a prevalence of TBI as high as 60 percent among adults with SCI (8, 9). In a single-center study, 31.6% of children with SCI had a concomitant brain injury (10). Other retrospective studies evaluating DD among patients with SCI who received inpatient rehabilitation excluded individuals under the age of 18 (8, 11, 12).

Co-occurring TBI and SCI can have considerable implications for a patient’s rehabilitation progress, speed of recovery, and prognosis (13). For example, it may impact one’s adjustment to disability, ability to learn new skills, motivation, tolerance of potentially sedating medications, and risk for complications. Moreover, the presence of DD may affect the speed of and the degree to which one recovers function after injury (4). Prior studies have demonstrated that adults with DD are more likely to require transfer from acute inpatient rehabilitation facilities to acute care facilities, more likely to suffer severe medical complications, and less likely to be discharged home as compared to those with SCI alone (11, 13). When compared to those with isolated TBI, adults with comorbid SCI demonstrate lower gains in cognitive domains during their courses of inpatient rehabilitation, particularly with problem solving and comprehension (12). When compared to adults with SCI without co-morbid TBI, those with DD are discharged from inpatient rehabilitation with greater cognitive impairment and having achieved less improvement in their motor Functional Independence Measure (FIM^™^) scores (14).

Anatomical factors contribute to the risk of DD in the pediatric population (15). Childrens’ heads are often disproportionately large and heavy relative to their bodies and are poorly supported by weak muscles and ligaments as well as unfused epiphyses (10). They also have increased water content within their intervertebral discs and shallow facet joints. All of these aspects contribute to a more malleable spine, increasing the risk of neurological injury even without bony injuries. Studies have shown that most spinal injuries in children occur at a higher location in the cervical spine, particularly at the C0-C2 level (16). These high cervical SCI levels are more likely to be associated with brain injury (17, 18).

Children’s skulls are thin and pliable in early development, providing less protection to the underlying brain (19, 20). As such, brain injuries can occur with or without an actual bony fracture; however, it has been suggested that the presence of skull fractures increases the possibility of underlying intracranial injury (21). Additionally, and uniquely to the pediatric population, as children’s heads grow, existing fractures subsequently grow and can result in delayed neurological deficits.

Key physiological differences exist between children and adults that may also have important implications for their risk for and recovery from DD. Blood volume is small by comparison, and cerebral blood flow varies with age. It is usually lowest at birth, peaks between ages 3 and 7, and progressively decreases to adult levels. Cerebral metabolism also changes with age. It starts at around 60% of adult values at birth and then it rapidly increases to values significantly greater than adult values by age 5. It subsequently slowly decreases to adult levels through adolescence. This is important for progressive myelination and synaptogenesis (22).

Pediatric DD also poses challenges due to the ongoing brain development that occurs in childhood. Cognitive impairments in children with brain injury may not be immediately evident after the injury, and may only become apparent as the child gets older (23). It may be particularly difficult to recognize mild TBI in younger children, though formal comprehensive testing may facilitate earlier detection of impairments in the cognitive domains (24).

This study was undertaken to better understand the epidemiology of DD in children in the United States. The primary aim was to establish the prevalence of DD with SCI and TBI in the pediatric population with secondary outcomes evaluating demographic data, length of stay, total hospital charges, and insurance status. Such analyses are critical to help better understand the needs of children with these injuries.

## Materials and methods

### Database

This study analyzed data from the KID Database, which consists of a compilation of de-identified discharge data from a sample of all hospital discharges of patients younger than 21 years of age, from 4,000 community, non-rehabilitation hospitals in the United States. It currently includes sites from 48 states and the District of Columbia. It is prepared every 3 years by the Healthcare Utilization Project (HCUP) of the Agency for Healthcare Research and Quality (25). It is used to identify, track, and analyze national trends in healthcare utilization, access, charges, quality, and outcomes. KID data elements include primary and secondary diagnoses and procedures, discharge status, patient demographics, hospital characteristics, expected payment source, total charges (Tot charge), length of stay (LOS), as well as severity and comorbidity measures (25). This study used de-identified data and was exempt from the University of Miami IRB review.

### Study design

A descriptive, retrospective, cross-sectional study was performed to assess the period prevalence (2016–2018) and epidemiology of SCI and combined SCI with TBI in pediatric groups. Age limit was set from 0 to 18 years. The International Classification of Diseases Version 10 (ICD-10) codes for SCI and TBI were included (see Appendix).

### Inclusion/exclusion criteria

All individuals in the database aged 0–18 years with a diagnosis of SCI made between 2016–2018 were included. Those with comorbid TBI were considered separately from those with isolated SCI.

### Statistical analysis

Normally distributed continuous variables (age) were compared with mean and standard deviation using independent sample *t*-tests. Initially, the LOS and Tot charge data sets were analyzed as normally distributed continuous variables, however, variances demonstrated that these sets were, in fact, not normally distributed (skewness values of 3.8 and 10.4, respectively). Thus, these non-normally distributed continuous variables (LOS, Tot charge) were compared with median and interquartile range using Wilcoxon signed-rank sum tests. Descriptive statistics for demographics and insurance status were generated. Categorical variables (race, gender, and insurance) were described with numbers and percentages and assessed using χ2 test.

Chi-Square test and *t*-test were conducted to examine differences in demographic characteristics by injury types to identify possible confounders. The Wilcoxon rank sums test was used to report the associations between specific injury and LOS or the respective total charge using a 2 tailed t approximation approach. Tot charges data were analyzed using a linear regression, adjusting for age, gender, race, and insurance status, to evaluate for the potential association of different types of injuries (SCI vs. DD). Data analysis was performed using Statistical Analysis System (SAS) version 9.4 and an α ≤ 0.05 was considered statistically significant.

## Results

### Prevalence

The database contained data on 1,286 children hospitalized with SCI during the time period of interest: 2016–2018. Utilizing the aforementioned ICD-10 codes for SCI and TBI, we identified 787 individuals with isolated SCI and 499 individuals with both SCI and TBI (Table 1). Thus, the prevalence of DD among children with SCI was 38.8% (95% CI 36.14,41.46%).

**Table 1:** Characteristics of pediatric patients with spinal cord injury alone and dual diagnosis.

	SCI alone (*n* = 787)	DD (*N* = 499)	*p*-value
Age (mean ± SD)	14.4 ± 4.3	13.2 ± 5.6	<0.0001
Gender (female; *n*, %)	288 (36.56)	166 (33.37)	0.22
Race, (*n*, %)			<0.0001
White	397 (50.44)	290 (58.12)	
Black	204 (25.92)	75 (15.03)	
Hispanic	125 (15.88)	87 (17.43)	
Other	61 (7.75)	47 (9.42)	
Insurance, (*n*, %)			0.44
Medicare/medicaid	359 (45.62)	212 (42.48)	
Private	350 (44.47)	240 (48.10)	
Other	78 (9.91)	47 (9.42)	
LOS (day), median (IQR)	4 (9)	6 (13)	0.0002
Total charges ($), Median (IQR)	$98.089 (186,215)	$124,198 (262,014)	0.0012

SCI, spinal cord injury; DD, dual diagnosis; LOS, length of stay; IQR, interquartile range.

### Age

**Figure 1:**
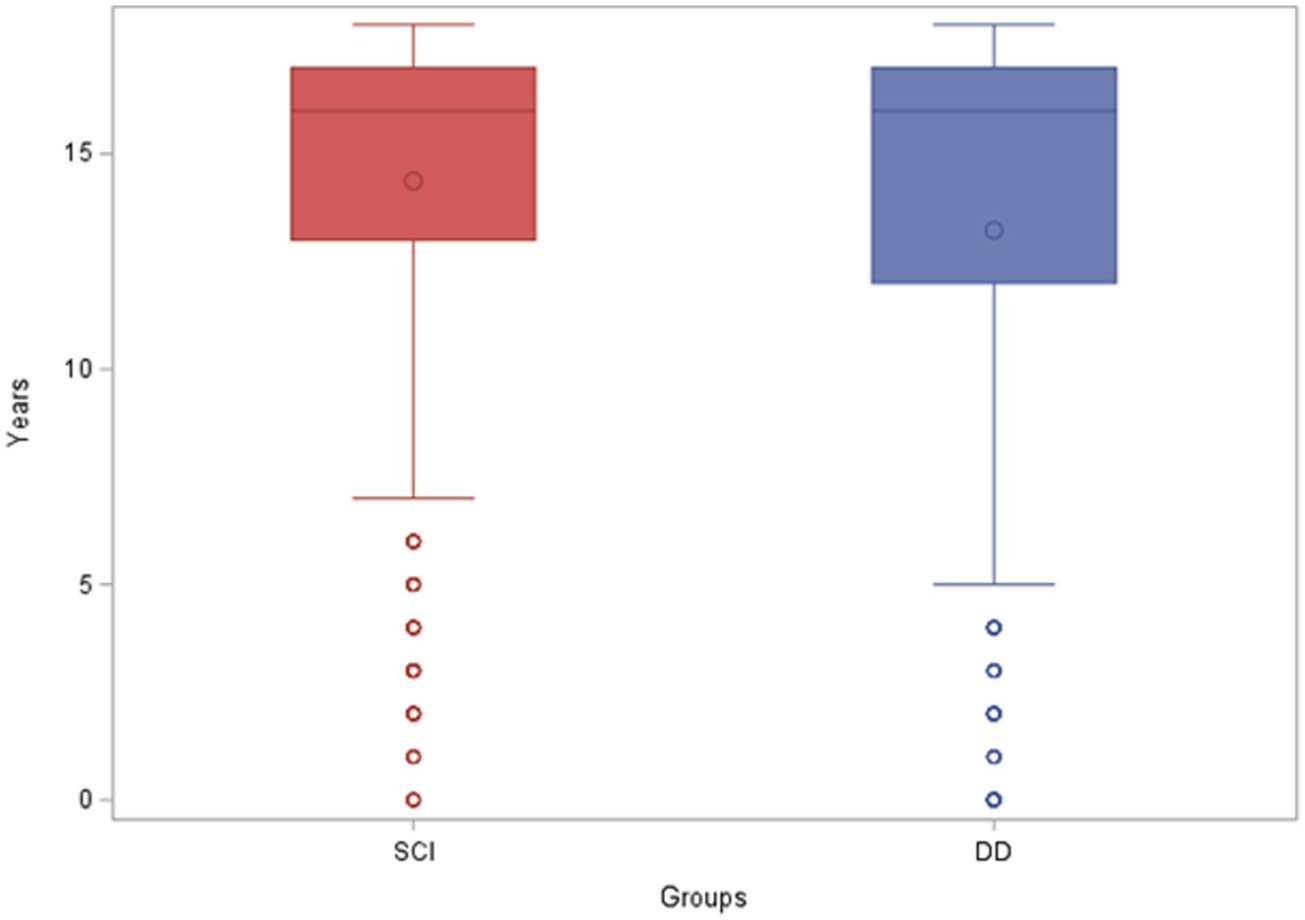
Age of pediatric patients with spinal cord injury alone and dual diagnosis. SCI, spinal cord injury; DD, dual diagnosis.

In the isolated SCI group, 75% of individuals were aged 13–17 (half of those were ages 16–17) and 25% between 0–13. In the DD group, 75% were 6–17 (half of those were ages 15–17) and 25% between the ages of 0–6. While the DD group demonstrated greater variability in the spread of ages, as indicated by an interquartile spread of 10 years (age 6–16) compared to 4 years (age 13–17) in the SCI group, both groups demonstrated a median age (16 and 15, respectively) in the upper quartile. This showed a clustering in the older teenage years (≥15) in both groups. The DD group also showed a clustering in the younger ages (<6 years), which was not observed in the isolated SCI group. As seen in Figure, those with SCI alone had a higher average age than DD and that difference was statistically significant (see Figure 1).

### Gender

In both groups, males comprised the largest demographic group, representing 64% and 67% of SCI and DD, respectively. The gender distribution between the two groups was not statistically significant (Table 1).

### Race

**Figure 2:**
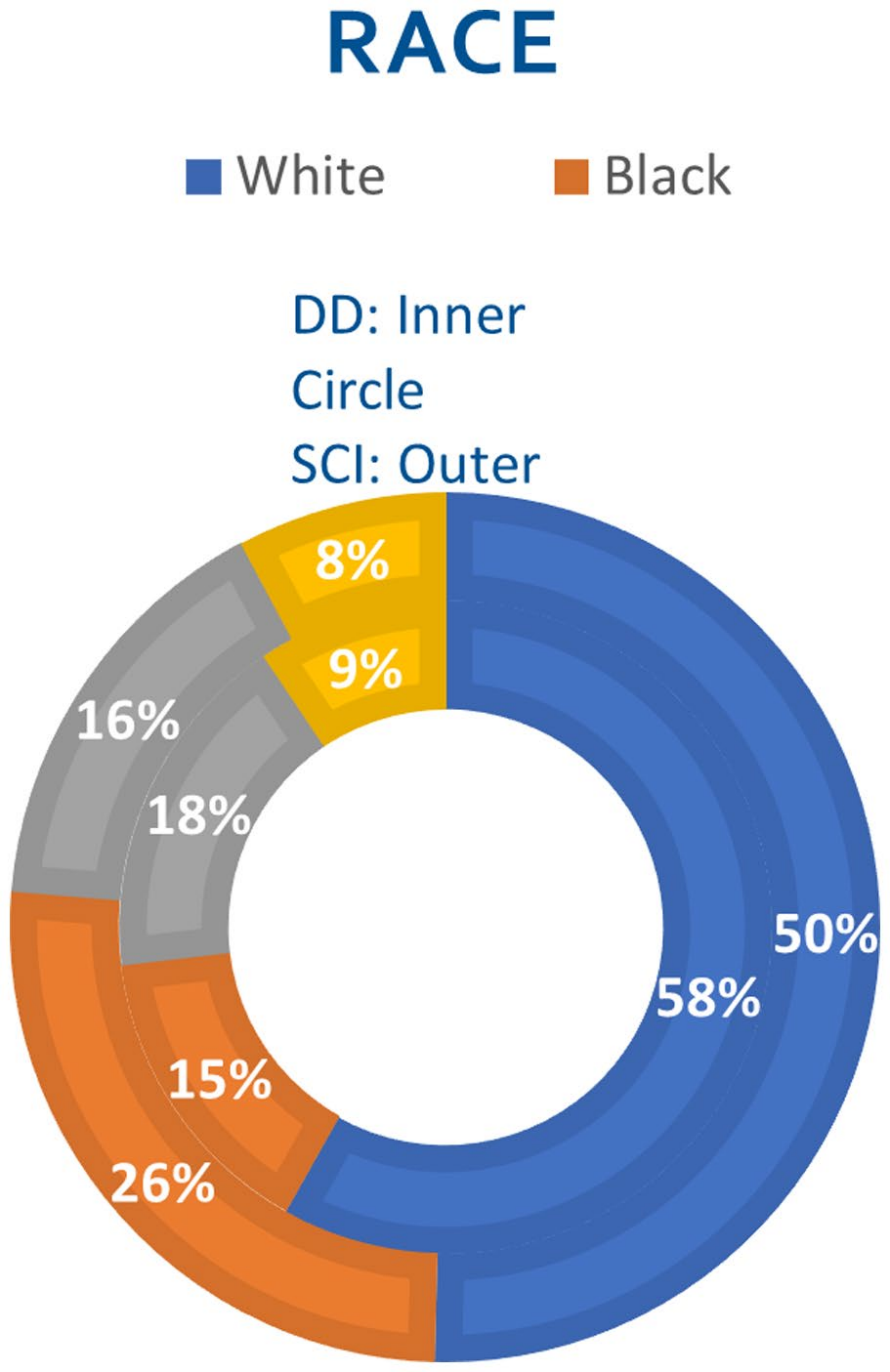
Race of pediatric patients with spinal cord injury alone and dual diagnosis. SCI, spinal cord injury; DD, dual diagnosis.

White people comprised the largest demographic population in both groups, representing 50% of the SCI and 58% of the DD group. This was followed by Black people and Hispanic people representing 26% and 16% of the SCI and 15% and 17% of the DD group, respectively. The race distribution between the two groups was statistically significant (Figure 2).

### Insurance

In the SCI group, 46% had Medicaid, 44% had private insurance, and 10% were listed as “other.” In the DD group, 42% had Medicaid, 48% had private insurance, and 10% were listed as “other.” We did not find any statistically significant differences in the type of insurance between the SCI alone group and DD group (Table 1).

### Length of stay and total charges

**Figure 3:**
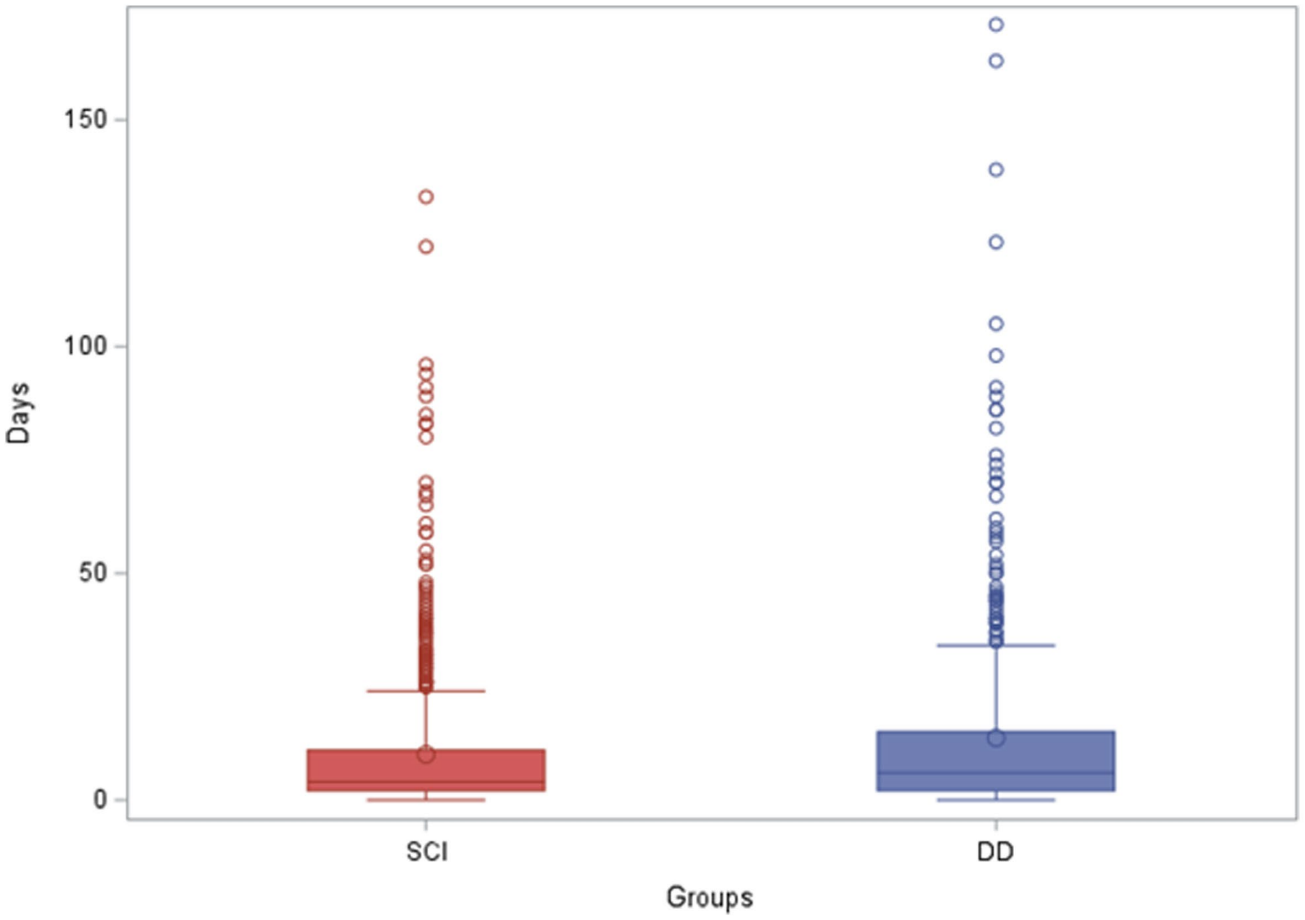
Length of stay of pediatric patients with spinal cord injury alone and dual diagnosis. LOS, length of stay; SCI, spinal cord injury; DD, dual diagnosis.

**Figure 4:**
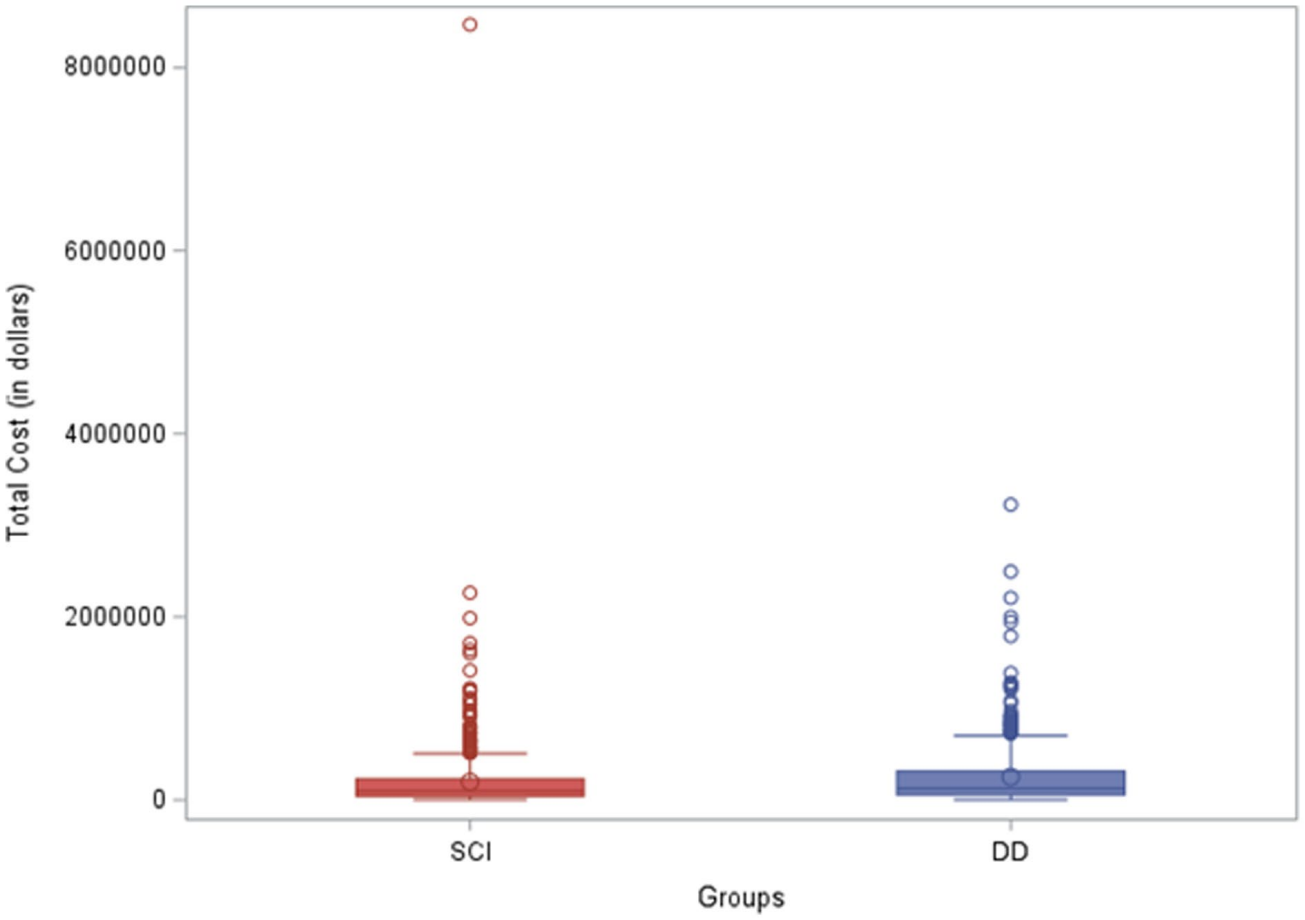
Total charges of pediatric patients with spinal cord injury alone and dual diagnosis. SCI, spinal cord injury; DD, dual diagnosis.

The average hospital LOS in the SCI group was 4 days, compared to 6 days in the DD group. The average total charge in the SCI group was $98,089 compared to the $124, 198 in the DD group. Two extra days of stay and approximately $25,000 extra charges per child in the DD group, compared to the SCI alone group, were statistically significant (Figures 3, 4). The same association and difference in extra charge persisted in the adjusted model.

## Discussion

While prior studies have highlighted the burden of traumatic SCI among US children and adolescents (26), published data on the prevalence of dual diagnosis in this population was previously limited to a single-center study (10).

To the best of our knowledge, this report represents the first epidemiological study comparing the two groups, SCI alone versus DD (i.e., SCI and TBI), in the pediatric population using a large representative national database. This study confirms the key finding of Vova et al. of a high prevalence of comorbid TBI among children with SCI. Accordingly, there is good reason to adopt practice guidelines that include assessment for TBI among all children hospitalized with SCI.

Another interesting finding of this study pertains to the distribution of age among children with SCI, both with and without comorbid TBI. Both groups demonstrated a clustering in the older teenage years. However, the DD group also showed greater variability in the younger ages and a small cluster in ages 6 and younger which was not seen in the isolated SCI group. SCI is relatively rare in children 15 years of age and younger; in fact, of the almost 2.4 million children identified through the KID Database in a 3-year span, SCI accounted for only 0.02% of the national data number. In contrast to the rare nature of SCI in the pediatric population, the 2015 Center for Disease Control (CDC) Report to Congress on TBI identified children aged 0–4 and adolescents aged 15–19 as a high-risk group for TBI (27). Our study also demonstrates that a younger population group is affected by concomitant TBI, in addition to the teenage population.

Race differences were observed in our study, with Caucasians comprising the largest demographic group and a greater percentage of the DD group than the isolated SCI group. This is similar to other pediatric studies which demonstrate that from 0–15 years of age, White people are more commonly found to have these injuries than Non-White people, with all modes of injury, except firearms (6). However, this differs from adult studies, which suggest that Non-White people make up the majority of SCI cases, due in large part to the elevated incidence among African-Americans.

Males represented a higher percentage than females (approximately 3:1 ratio) in both the isolated SCI and DD groups. However, amongst those with SCI, neither gender was statistically more likely to have comorbid TBI. According to the 2011 National Spinal Cord Injury Statistical Center, Birmingham, Alabama data, the ratio of males to females in the SCI population alone is approximately 4:1 (28). There is limited data on the ratio of males to females in the DD population, with one study finding approximately a 1.8:1 M:F ratio (12). While males comprise the majority in both the adult and pediatric SCI and DD population, in the pediatric population, the gap between the genders is narrowed.

This study also revealed that pediatric DD is associated with longer hospital lengths of stay and higher health expenditures when compared to the same population with isolated SCI. According to Zonfrillo et al., children hospitalized with severe TBI and SCI did not demonstrate a difference in standardized hospital costs relative to their home zip code level median annual household income (29). In this study, the type of insurance was similar between the SCI alone group and DD group. In both groups, there were a relatively equal percentage of those with Medicaid and those with private insurance. However, we observed that the length of stay and total hospital cost in the DD group was longer and costlier than in the SCI alone group. Consequently, having a DD places a higher burden on the healthcare system. Identifying these increased healthcare costs helps to suggest improvement in allocation of resources.

Among the limitations of this study is the potential for information bias. Similar to what Sikka et al. observed in 2019, in attempting to determine prevalence of TBI from acute care records, documentation variability exists among physicians and advanced practitioners (30). Additionally, the analysis relies on administrative billing data for the identification of cases where the accuracy of the codes may be unreliable. This likely results in under-representation of cases, suggesting that the percentage of dual diagnosis is probably higher than found. Moreover, ICD-10 codes do not reflect or capture the degree of brain injury severity. Also, only data pertaining to the acute hospitalization was available, and there was no information on rehabilitation nor any outcomes after hospital discharge. Additionally, because the data excluded deaths prior to admission, we could not evaluate the prevalence of DD among those with injuries resulting in death at the scene.

A final limitation concerns its generalizability to a global pediatric population. While some demographic data (e.g., gender and age) is likely widely generalizable, we do not believe one can appropriately extrapolate U.S. data regarding insurance status, race, hospital charges, or hospital lengths of stay to draw conclusions about these variables among injured children in other countries.

## Conclusion

This study has demonstrated that more than a third of U.S. children hospitalized with SCI have comorbid TBI. DD among children contributes to longer hospital lengths of stay and greater health care expenditures when compared to SCI alone. Greater awareness of DD in children is needed to ensure appropriate screening for TBI in pediatric patients with SCI.

To better identify the true prevalence of dual diagnosis in children, it would be beneficial to prospectively collect data in those with SCI that includes comprehensive evaluation for TBI. Such evaluations would need to include neurological imaging reports, Glasgow Coma Scale scores, the presence and/or duration of loss of consciousness and post-traumatic amnesia, and the results of neuropsychological testing. Further research is also necessary to identify the impact of DD on the functional outcomes and quality of life of affected children, as well as their risks for mortality and long-term complications.

## Data availability statement

The data analyzed in this study was obtained from the Agency of Healthcare Research and Quality (AHRQ), Healthcare Cost and Utilization Project (HCUP), Kid’s Inpatient Database (KID; https://hcup-us.ahrq.gov/kidoverview.jsp), the following licenses/restrictions apply: all HCUP data users, including data purchasers and collaborators, must complete the online HCUP Data Use Agreement Training Tool, and must read and sign the Data Use Agreement for Nationwide Databases before HCUP data can be accessed. Requests to access these datasets should be directed to HCUP, hcup@ahrq.gov.

## Ethics statement

Ethical review and approval was not required for the study on human participants in accordance with the local legislation and institutional requirements. Written informed consent from the patients/participants or patients/participants’ legal guardian/next of kin was not required to participate in this study in accordance with the national legislation and the institutional requirements.

## Author contributions

JG made substantial contributions to the conception and design of the work, the acquisition, analysis, and interpretation of the data, drafted the work and substantively revised it, and has approved the submitted version. LS made substantial contributions to the design of the work, the acquisition, analysis, and interpretation of the data, substantively revised the work, and has approved the submitted version. ET made substantial contributions to the interpretation of the data and has approved the submitted version. NR made substantial contributions to the acquisition, analysis, and interpretation of the data and has approved the submitted version. CB made substantial contributions to the acquisition, analysis, and interpretation of the data and has approved the submitted version. KL made substantial contributions to the conception and design of the work, the acquisition, analysis, and interpretation of the data, and has approved the submitted version. AV made substantial contributions to the conception and design of the work, the acquisition, analysis, and interpretation of the data, drafted the work and substantively revised it, and has approved the submitted version. All agree to be accountable for all aspects of the work in ensuring that questions related to the accuracy or integrity of any part of the work are appropriately investigated and resolved. All authors contributed to the article and approved the submitted version.
