# Supplementary material for: Dual diagnosis of TBI and SCI: an epidemiological study in the pediatric population
Source: Front Neurol. 2023 Sep 27;14:1241550. doi: 10.3389/fneur.2023.1241550 (PMC10565222; doi:10.3389/fneur.2023.1241550)
Supplement: Supplementary file 1 [file Table_1.docx]

Appendix, Table 1: Spinal Cord Injury ICD 10 Codes and Definitions

| ICD10 Code | Definition |
| --- | --- |
| S12.000A | Unspecified displaced fracture of first cervical vertebra, initial encounter for closed fracture |
| S12.001A | Unspecified nondisplaced fracture of first cervical vertebra, initial encounter for closed fracture |
| S12.100A | Unspecified displaced fracture of second cervical vertebra, initial encounter for closed fracture |
| S12.101A | Unspecified nondisplaced fracture of second cervical vertebra, initial encounter for closed fracture |
| S12.200A | Unspecified displaced fracture of third cervical vertebra, initial encounter for closed fracture |
| S12.201A | Unspecified nondisplaced fracture of third cervical vertebra, initial encounter for closed fracture |
| S12.300A | Unspecified displaced fracture of fourth cervical vertebra, initial encounter for closed fracture |
| S12.301A | Unspecified nondisplaced fracture of fourth cervical vertebra, initial encounter for closed fracture |
| S12.400A | Unspecified displaced fracture of fifth cervical vertebra, initial encounter for closed fracture |
| S12.401A | Unspecified nondisplaced fracture of fifth cervical vertebra, initial encounter for closed fracture |
| S12.500A | Unspecified displaced fracture of sixth cervical vertebra, initial encounter for closed fracture |
| S12.501A | Unspecified nondisplaced fracture of sixth cervical vertebra, initial encounter for closed fracture |
| S12.600A | Unspecified displaced fracture of seventh cervical vertebra, initial encounter for closed fracture |
| S12.601A | Unspecified nondisplaced fracture of seventh cervical vertebra, initial encounter for closed fracture |
| S14.101A | Unspecified injury at C1 level of cervical spinal cord, initial encounter |
| S14.102A | Unspecified injury at C2 level of cervical spinal cord, initial encounter |
| S14.103A | Unspecified injury at C3 level of cervical spinal cord, initial encounter |
| S14.104A | Unspecified injury at C4 level of cervical spinal cord, initial encounter |
| S14.105A | Unspecified injury at C5 level of cervical spinal cord, initial encounter |
| S14.106A | Unspecified injury at C6 level of cervical spinal cord, initial encounter |
| S14.107A | Unspecified injury at C7 level of cervical spinal cord, initial encounter |
| S14.108A | Unspecified injury at C8 level of cervical spinal cord, initial encounter |
| S14.109A | Unspecified injury at unspecified level of cervical spinal cord, initial encounter |
| S14.111A | Complete lesion at C1 level of cervical spinal cord, initial encounter |
| S14.112A | Complete lesion at C2 level of cervical spinal cord, initial encounter |
| S14.113A | Complete lesion at C3 level of cervical spinal cord, initial encounter |
| S14.114A | Complete lesion at C4 level of cervical spinal cord, initial encounter |
| S14.115A | Complete lesion at C5 level of cervical spinal cord, initial encounter |
| S14.116A | Complete lesion at C6 level of cervical spinal cord, initial encounter |
| S14.117A | Complete lesion at C7 level of cervical spinal cord, initial encounter |
| S14.118A | Complete lesion at C8 level of cervical spinal cord, initial encounter |
| S14.121A | Central cord syndrome at C1 level of cervical spinal cord, initial encounter |
| S14.122A | Central cord syndrome at C2 level of cervical spinal cord, initial encounter |
| S14.123A | Central cord syndrome at C3 level of cervical spinal cord, initial encounter |
| S14.124A | Central cord syndrome at C4 level of cervical spinal cord, initial encounter |
| S14.125A | Central cord syndrome at C5 level of cervical spinal cord, initial encounter |
| S14.126A | Central cord syndrome at C6 level of cervical spinal cord, initial encounter |
| S14.127A | Central cord syndrome at C7 level of cervical spinal cord, initial encounter |
| S14.128A | Central cord syndrome at C8 level of cervical spinal cord, initial encounter |
| S14.131A | Anterior cord syndrome at C1 level of cervical spinal cord, initial encounter |
| S14.132A | Anterior cord syndrome at C2 level of cervical spinal cord, initial encounter |
| S14.133A | Anterior cord syndrome at C3 level of cervical spinal cord, initial encounter |
| S14.134A | Anterior cord syndrome at C4 level of cervical spinal cord, initial encounter |
| S14.135A | Anterior cord syndrome at C5 level of cervical spinal cord, initial encounter |
| S14.136A | Anterior cord syndrome at C6 level of cervical spinal cord, initial encounter |
| S14.137A | Anterior cord syndrome at C7 level of cervical spinal cord, initial encounter |
| S14.138A | Anterior cord syndrome at C8 level of cervical spinal cord, initial encounter |
| S14.151A | Other incomplete lesion at C1 level of cervical spinal cord, initial encounter |
| S14.152A | Other incomplete lesion at C2 level of cervical spinal cord, initial encounter |
| S14.153A | Other incomplete lesion at C3 level of cervical spinal cord, initial encounter |
| S14.154A | Other incomplete lesion at C4 level of cervical spinal cord, initial encounter |
| S14.155A | Other incomplete lesion at C5 level of cervical spinal cord, initial encounter |
| S14.156A | Other incomplete lesion at C6 level of cervical spinal cord, initial encounter |
| S14.157A | Other incomplete lesion at C7 level of cervical spinal cord, initial encounter |
| S14.158A | Other incomplete lesion at C8 level of cervical spinal cord, initial encounter |
| S24.101A | Unspecified injury at T1 level of thoracic spinal cord, initial encounter |
| S24.102A | Unspecified injury at T2-T6 level of thoracic spinal cord, initial encounter |
| S24.103A | Unspecified injury at T7-T10 level of thoracic spinal cord, initial encounter |
| S24.104A | Unspecified injury at T11-T12 level of thoracic spinal cord, initial encounter |
| S24.109 | Unspecified injury at unspecified level of thoracic spinal cord |
| S24.111A | Complete lesion at T1 level of thoracic spinal cord, initial encounter |
| S24.112A | Complete lesion at T2-T6 level of thoracic spinal cord, initial encounter |
| S24.113A | Complete lesion at T7-T10 level of thoracic spinal cord, initial encounter |
| S24.114A | Complete lesion at T11-T12 level of thoracic spinal cord, initial encounter |
| S24.131A | Anterior cord syndrome at T1 level of thoracic spinal cord, initial encounter |
| S24.132A | Anterior cord syndrome at T2-T6 level of thoracic spinal cord, initial encounter |
| S24.133A | Anterior cord syndrome at T7-T10 level of thoracic spinal cord, initial encounter |
| S24.134A | Anterior cord syndrome at T11-T12 level of thoracic spinal cord, initial encounter |
| S24.151A | Other incomplete lesion at T1 level of thoracic spinal cord, initial encounter |
| S24.152A | Other incomplete lesion at T2-T6 level of thoracic spinal cord, initial encounter |
| S24.153A | Other incomplete lesion at T7-T10 level of thoracic spinal cord, initial encounter |
| S24.154A | Other incomplete lesion at T11-T12 level of thoracic spinal cord, initial encounter |
| S34.109A | Unspecified injury to unspecified level of lumbar spinal cord, initial encounter |
| S34.139A | Unspecified injury to sacral spinal cord, initial encounter |
| S34.3XXA | Injury of cauda equina, initial encounter |
