# Supplementary material for: Dual diagnosis of TBI and SCI: an epidemiological study in the pediatric population
Source: Front Neurol. 2023 Sep 27;14:1241550. doi: 10.3389/fneur.2023.1241550 (PMC10565222; doi:10.3389/fneur.2023.1241550)
Supplement: Supplementary file 2 [file Table_2.docx]

Appendix, Table 2: Traumatic Brain Injury ICD 10 Codes and Definitions

| ICD10 Code | Definition |
| --- | --- |
| S01.90XA | Unspecified open wound of unspecified part of head, initial encounter |
| S06.0X0A | Concussion without loss of consciousness, initial encounter |
| S06.0X1A | Concussion with loss of consciousness of 30 minutes or less, initial encounter |
| S06.0X9A | Concussion with loss of consciousness of unspecified duration, initial encounter |
| S06.1X0A | Traumatic cerebral edema without loss of consciousness, initial encounter |
| S06.1X1A | Traumatic cerebral edema with loss of consciousness of 30 minutes or less, initial encounter |
| S06.1X2A | Traumatic cerebral edema with loss of consciousness of 31 minutes to 59 minutes, initial encounter |
| S06.1X3A | Traumatic cerebral edema with loss of consciousness of 1 hour to 5 hours 59 minutes, initial encounter |
| S06.1X4A | Traumatic cerebral edema with loss of consciousness of 6 hours to 24 hours, initial encounter |
| S06.1X5A | Traumatic cerebral edema with loss of consciousness greater than 24 hours with return to pre-existing conscious level, initial encounter |
| S06.1X6A | Traumatic cerebral edema with loss of consciousness greater than 24 hours without return to pre-existing conscious level with patient surviving, initial encounter |
| S06.1X7A | Traumatic cerebral edema with loss of consciousness of any duration with death due to brain injury prior to regaining consciousness, initial encounter |
| S06.1X8A | Traumatic cerebral edema with loss of consciousness of any duration with death due to other cause prior to regaining consciousness, initial encounter |
| S06.1X9A | Traumatic cerebral edema with loss of consciousness of unspecified duration, initial encounter |
| S06.330A | Contusion and laceration of cerebrum, unspecified, without loss of consciousness, initial encounter |
| S06.331A | Contusion and laceration of cerebrum, unspecified, with loss of consciousness of 30 minutes or less, initial encounter |
| S06.332A | Contusion and laceration of cerebrum, unspecified, with loss of consciousness of 31 minutes to 59 minutes, initial encounter |
| S06.333A | Contusion and laceration of cerebrum, unspecified, with loss of consciousness of 1 hour to 5 hours 59 minutes, initial encounter |
| S06.334A | Contusion and laceration of cerebrum, unspecified, with loss of consciousness of 6 hours to 24 hours, initial encounter |
| S06.335A | Contusion and laceration of cerebrum, unspecified, with loss of consciousness greater than 24 hours with return to pre-existing conscious level, initial encounter |
| S06.336A | Contusion and laceration of cerebrum, unspecified, with loss of consciousness greater than 24 hours without return to pre-existing conscious level with patient surviving, initial encounter |
| S06.337A | Contusion and laceration of cerebrum, unspecified, with loss of consciousness of any duration with death due to brain injury prior to regaining consciousness, initial encounter |
| S06.360A | Traumatic hemorrhage of cerebrum, unspecified, without loss of consciousness, initial encounter |
| S06.361A | Traumatic hemorrhage of cerebrum, unspecified, with loss of consciousness of 30 minutes or less, initial encounter |
| S06.362A | Traumatic hemorrhage of cerebrum, unspecified, with loss of consciousness of 31 minutes to 59 minutes, initial encounter |
| S06.363A | Traumatic hemorrhage of cerebrum, unspecified, with loss of consciousness of 1 hours to 5 hours 59 minutes, initial encounter |
| S06.364A | Traumatic hemorrhage of cerebrum, unspecified, with loss of consciousness of 6 hours to 24 hours, initial encounter |
| S06.365A | Traumatic hemorrhage of cerebrum, unspecified, with loss of consciousness greater than 24 hours with return to pre-existing conscious level, initial encounter |
| S06.366A | Traumatic hemorrhage of cerebrum, unspecified, with loss of consciousness greater than 24 hours without return to pre-existing conscious level with patient surviving, initial encounter |
| S06.367A | Traumatic hemorrhage of cerebrum, unspecified, with loss of consciousness of any duration with death due to brain injury prior to regaining consciousness, initial encounter |
| S06.368A | Traumatic hemorrhage of cerebrum, unspecified, with loss of consciousness of any duration with death due to other cause prior to regaining consciousness, initial encounter |
| S06.369A | Traumatic hemorrhage of cerebrum, unspecified, with loss of consciousness of unspecified duration, initial encounter |
| S06.370A | Contusion, laceration, and hemorrhage of cerebellum without loss of consciousness, initial encounter |
| S06.380A | Contusion, laceration, and hemorrhage of brainstem without loss of consciousness, initial encounter |
| S06.4X0A | Epidural hemorrhage without loss of consciousness, initial encounter |
| S06.4X1A | Epidural hemorrhage with loss of consciousness of 30 minutes or less, initial encounter |
| S06.4X2A | Epidural hemorrhage with loss of consciousness of 31 minutes to 59 minutes, initial encounter |
| S06.4X3A | Epidural hemorrhage with loss of consciousness of 1 hour to 5 hours 59 minutes, initial encounter |
| S06.4X4A | Epidural hemorrhage with loss of consciousness of 6 hours to 24 hours, initial encounter |
| S06.4X5A | Epidural hemorrhage with loss of consciousness greater than 24 hours with return to pre-existing conscious level, initial encounter |
| S06.4X6A | Epidural hemorrhage with loss of consciousness greater than 24 hours without return to pre-existing conscious level with patient surviving, initial encounter |
| S06.4X7A | Epidural hemorrhage with loss of consciousness of any duration with death due to brain injury prior to regaining consciousness, initial encounter |
| S06.4X8A | Epidural hemorrhage with loss of consciousness of any duration with death due to other causes prior to regaining consciousness, initial encounter |
| S06.4X9A | Epidural hemorrhage with loss of consciousness of unspecified duration, initial encounter |
| S06.5X0A | Traumatic subdural hemorrhage without loss of consciousness, initial encounter |
| S06.5X1A | Traumatic subdural hemorrhage with loss of consciousness of 30 minutes or less, initial encounter |
| S06.5X2A | Traumatic subdural hemorrhage with loss of consciousness of 31 minutes to 59 minutes, initial encounter |
| S06.5X3A | Traumatic subdural hemorrhage with loss of consciousness of 1 hour to 5 hours 59 minutes, initial encounter |
| S06.5X4A | Traumatic subdural hemorrhage with loss of consciousness of 6 hours to 24 hours, initial encounter |
| S06.5X5A | Traumatic subdural hemorrhage with loss of consciousness greater than 24 hours with return to pre-existing conscious level, initial encounter |
| S06.5X6A | Traumatic subdural hemorrhage with loss of consciousness greater than 24 hours without return to pre-existing conscious level with patient surviving, initial encounter |
| S06.5X7A | Traumatic subdural hemorrhage with loss of consciousness of any duration with death due to brain injury before regaining consciousness, initial encounter |
| S06.5X8A | Traumatic subdural hemorrhage with loss of consciousness of any duration with death due to other cause before regaining consciousness, initial encounter |
| S06.5X9A | Traumatic subdural hemorrhage with loss of consciousness of unspecified duration, initial encounter |
| S06.6X0A | Traumatic subarachnoid hemorrhage without loss of consciousness, initial encounter |
| S06.6X1A | Traumatic subarachnoid hemorrhage with loss of consciousness of 30 minutes or less, initial encounter |
| S06.6X2A | Traumatic subarachnoid hemorrhage with loss of consciousness of 31 minutes to 59 minutes, initial encounter |
| S06.6X3A | Traumatic subarachnoid hemorrhage with loss of consciousness of 1 hour to 5 hours 59 minutes, initial encounter |
| S06.6X4A | Traumatic subarachnoid hemorrhage with loss of consciousness of 6 hours to 24 hours, initial encounter |
| S06.6X5A | Traumatic subarachnoid hemorrhage with loss of consciousness greater than 24 hours with return to pre-existing conscious level, initial encounter |
| S06.6X6A | Traumatic subarachnoid hemorrhage with loss of consciousness greater than 24 hours without return to pre-existing conscious level with patient surviving, initial encounter |
| S06.6X7A | Traumatic subarachnoid hemorrhage with loss of consciousness of any duration with death due to brain injury prior to regaining consciousness, initial encounter |
| S06.6X8A | Traumatic subarachnoid hemorrhage with loss of consciousness of any duration with death due to other cause prior to regaining consciousness, initial encounter |
| S06.6X9A | Traumatic subarachnoid hemorrhage with loss of consciousness of unspecified duration, initial encounter |
| S06.890A | Other specified intracranial injury without loss of consciousness, initial encounter |
| S06.891A | Other specified intracranial injury with loss of consciousness of 30 minutes or less, initial encounter |
| S06.892A | Other specified intracranial injury with loss of consciousness of 31 minutes to 59 minutes, initial encounter |
| S06.893A | Other specified intracranial injury with loss of consciousness of 1 hour to 5 hours 59 minutes, initial encounter |
| S06.894A | Other specified intracranial injury with loss of consciousness of 6 hours to 24 hours, initial encounter |
| S06.895A | Other specified intracranial injury with loss of consciousness greater than 24 hours with return to pre-existing conscious level, initial encounter |
| S06.896A | Other specified intracranial injury with loss of consciousness greater than 24 hours without return to pre-existing conscious level with patient surviving, initial encounter |
| S06.897A | Other specified intracranial injury with loss of consciousness of any duration with death due to brain injury prior to regaining consciousness, initial encounter |
| S06.898A | Other specified intracranial injury with loss of consciousness of any duration with death due to other cause prior to regaining consciousness, initial encounter |
| S06.899A | Other specified intracranial injury with loss of consciousness of unspecified duration, initial encounter |
| S06.9X0A | Unspecified intracranial injury without loss of consciousness, initial encounter |
| S06.9X1A | Unspecified intracranial injury with loss of consciousness of 30 minutes or less, initial encounter |
| S06.9X2A | Unspecified intracranial injury with loss of consciousness of 31 minutes to 59 minutes, initial encounter |
| S06.9X3A | Unspecified intracranial injury with loss of consciousness of 1 hour to 5 hours 59 minutes, initial encounter |
| S06.9X4A | Unspecified intracranial injury with loss of consciousness of 6 hours to 24 hours, initial encounter |
| S06.9X5A | Unspecified intracranial injury with loss of consciousness greater than 24 hours with return to pre-existing conscious level, initial encounter |
| S06.9X6A | Unspecified intracranial injury with loss of consciousness greater than 24 hours without return to pre-existing conscious level with patient surviving, initial encounter |
| S06.9X9A | Unspecified intracranial injury with loss of consciousness of unspecified duration, initial encounter |
| S09.8XXA | Other specified injuries of head, initial encounter |
| S09.90XA | Unspecified injury of head, initial encounter |
